# Institutional partnerships enable long-term use of Earth observations in water security and resilience

**DOI:** 10.1038/s44458-026-00178-9

**Published:** 2026-11-09

**Authors:** Chinmay Deval, Eric Anderson, Ashutosh Limaye, Daniel Irwin, Pete Epanchin

**Affiliations:** 1 The Lab for Applied Science, Earth System Science Center, The University of Alabama in Huntsville, Huntsville, AL USA; 2 Earth and Planetary Science Office, NASA Marshall Space Flight Center, Huntsville, AL USA; 3 Spatial Informatics Group Natural Assets Laboratory (Sig Nal), Pleasanton, CA USA

**Keywords:** Hydrology, Water resources, Developing world, Sustainability, Climate-change adaptation

## Abstract

Global water security demands reliable monitoring and resilient infrastructure, especially amid global and regional change. Although Earth observation technologies provide unprecedented information, institutions responsible for water, environmental, and disaster risk management in low- and middle-income countries still underuse their potential. Long-term use of Earth observations depends on translational infrastructure that connects scientific capabilities with operational decision-making. The SERVIR initiative provides a 20-year record (2005-2025) for identifying mechanisms that enable Earth observations to become embedded in policy and practice. Analysis of this record suggests that long-term use of Earth observations depends on distributed boundary organizations, iterative service co-design and embedded human capacity. Cases in which Earth observations remained in use after donor funding ended highlight transferable design principles for future digital public goods and show that long-term adaptation requires governance systems that bridge science and policy through locally driven co-production.

## Abstract (web summary)

Earth observations can support water security and resilience, but durable use depends on institutional arrangements rather than technology transfer alone. Lessons from 20 years of the SERVIR initiative highlight mechanisms that integrate Earth observations into decision-making.

## Earth Observations Remain Underused in Water Governance

Accelerating global environmental change is affecting the way societies understand and govern water^1^. Droughts, floods, and water contamination have already disrupted livelihoods for billions, while the demand for fresh water continues to rise. The achievement of the Sustainable Development Goals (SDG)—particularly SDG 6 and SDG 13—depends on the ability of governments’ and communities’ to transform data into decisions that improve resilience. However, despite the rapid scientific advances in satellite monitoring, hydrological modeling, and weather forecasting^2–6^, a persistent “usability gap” continues to separate global scientific advances from the local institutions and communities most vulnerable to environmental and weather-related impacts^7–10^. The barrier is not a lack of data, but a lack of mechanisms that translate science into governance.

Over the past two decades, researchers and practitioners have emphasized the importance of co-development, participatory knowledge systems, and “boundary organizations” that bridge science and policy^11–15^. More recently, sustainability science has begun to examine how these collaborative practices become institutionalized within organizations and governance systems to sustain long-term societal impact^16,17^. These concepts recognize that sustainable adaptation requires iterative learning between knowledge producers and users. Moving beyond ad hoc projects requires institutionalizing these practices through “translational infrastructures”—boundary systems that systematically align scientific capabilities with operational mandates.

SERVIR—an initiative co-led by NASA and USAID—provides a 20-year empirical record of this principle operating on a global scale^18,19^. From 2005, when it was established, to 2025, when the support from the US government (NASA and USAID) concluded, SERVIR operated as a distributed network of regional “hubs”, each hosted by leading geospatial or environmental institutions across Africa, Asia, and Latin America. Through these hubs, this network linked NASA’s Earth observation (EO) capabilities with national and regional agencies, academic institutions, and civil society organizations that design and implement water and related local and regional resilience policies.

**Fig. 1:**
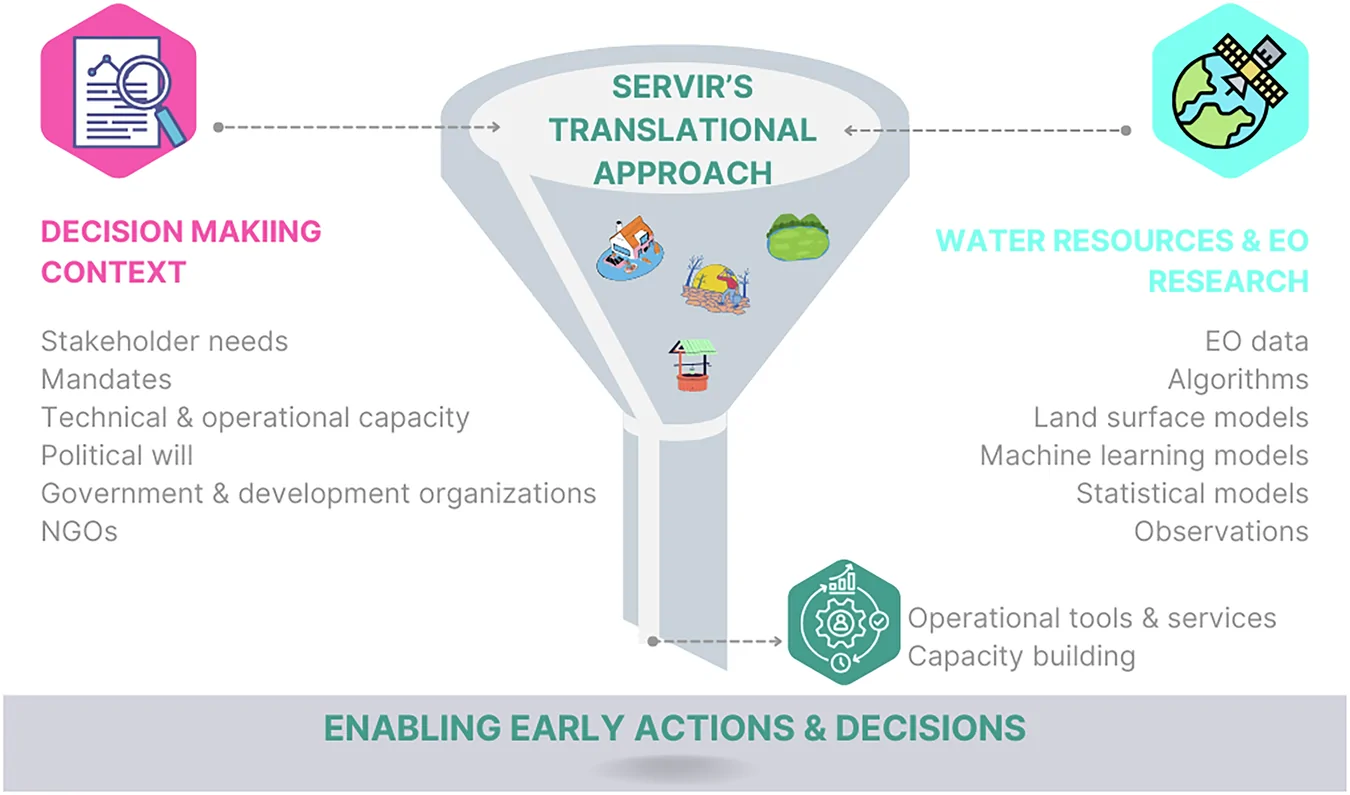
A translational model connecting Earth observation science and decision-making contexts. Translational infrastructures act as a boundary system linking water resources research—spanning satellite-based Earth observations, models, and algorithms—with real-world decision contexts shaped by mandates, capacity, and political will. Through iterative co-development, these networks translate global data into operational tools and services that enable anticipatory actions and informed policy decisions.

This institutional arrangement differed fundamentally from traditional technology transfer programs. Rather than exporting predesigned scientific products, it worked with local partners with mandates from national governments in their regions to co-identify problems, co-develop tools, and build sustained technical and institutional capacity (Fig. 1). Its Service Planning Approach (SPA) guided this process through a sequence of consultation, needs assessment, service design, and iterative refinement. This model positioned hubs as a network of boundary organizations operating simultaneously within the scientific, policy, and development ecosystems. It turned EO science into a process of learning, negotiation, action and decision-making, not simply data delivery.

By treating this 20-year operational record as an evolving system rather than a static program, this paper contributes to sustainability-transitions research. We interpret these collaborative networks as a form of sustainability-transition infrastructure—a socio-technical system that enabled the translation of global data into locally actionable knowledge. Drawing on two decades of operational experience across diverse regions in LMICs, we explore three overarching questions:

1. What conditions enable sustained uptake of EO-based science for resilience in the face of global and regional environmental change?
2. How does co-development strengthen institutional learning and policy alignment?
3. What design principles from this empirical record can inform future applied EO initiatives?

To address these questions, we synthesize insights from the network’s implementation across multiple water-related domains. Although it is important to note that SERVIR operated in five thematic areas, namely Water Security, Agriculture and Food Security, Ecosystem and Carbon Management, Weather and Climate Resilience, and Air Quality and Health. Rather than cataloging technical details, we analyze cross-cutting mechanisms that link scientific credibility, stakeholder legitimacy, and governance salience—the “triad” often identified as essential for actionable knowledge^11^. While recent literature (e.g., see ref. ^18^) comprehensively details the technical EO tools and hydrologic applications developed across these networks, this perspective isolates the institutional arrangements and boundary-spanning mechanisms required to make those tools actionable. In doing so, we align experience with broader debates in sustainability science on how to operationalize co-development for resilient societies.

The paper proceeds in five sections. First, we outline the conceptual framework underpinning this co-development model, situating it within sustainability transitions theory. Next, we synthesize evidence from practice, clustering examples by their adaptation function: anticipatory action, water resilience, and environmental quality. We then discuss the institutional mechanisms that enabled sustained impact, including trust networks, embedded partnerships, and adaptive governance. The final sections explore future pathways for digital public goods and global adaptation systems, concluding with generalizable design principles for planetary resilience.

The contribution of this perspective is not another description of co-development, but the identification of four interacting institutional capabilities that enable Earth observations to become embedded in long-term sustainability decision-making. We argue that these capabilities—boundary organizations, structured co-development, translational expertise, and adaptive learning—constitute a transferable institutional architecture applicable well beyond SERVIR.

## Institutions Make Earth Observations Actionable

Over the past decade, sustainability science has increasingly shifted from viewing scientific knowledge generation as the primary challenge to understanding how knowledge becomes embedded within governance, institutions, and decision-making systems^8,16,17^. Effective adaptation therefore requires systems that link knowledge, governance, and action through continuous learning. This has given rise to the concept of knowledge co-development—the collaborative process by which scientists, policymakers, and communities jointly define problems, design solutions, and evaluate outcomes^11–13^. Co-development redefines the role of science from delivering answers to facilitating deliberation. Within this literature, boundary organizations play a vital role as intermediaries that connect scientific and political domains, ensuring both scientific credibility and policy relevance^20^.

SERVIR provides a rare long-term empirical example of how these concepts can be operationalized through sustained institutional partnerships. Its distinguishing feature was the systematic institutionalization of co-development through a structured operational model that combined global scientific assets with regional governance networks^18^. We refer to this model as sustainability infrastructure: a socio-technical system designed to make scientific knowledge actionable, transparent, and durable across contexts. SERVIR’s infrastructure consisted of four interlocking components—each corresponding to a stage in the co-development process.

### Boundary architecture: the hub-and-network model

SERVIR’s regional hubs were hosted within established local institutions—such as research centers, regional commissions, and universities—that already possessed legitimacy with governments and communities. These hubs acted as conveners, mediating between NASA scientists, national agencies, and end users. They localized scientific expertise, translated technical language, and built trust through repeated engagement. The network architecture—spanning hubs in the Hindu Kush Himalaya, East and Southern Africa, West Africa, the Mekong, Central America, and the Amazon—enabled peer learning and transboundary exchange. This distributed boundary structure transformed SERVIR from a project into a long-term system for co-developing EO driven information services.

### The service planning approach: from data to decision

**Fig. 2:**
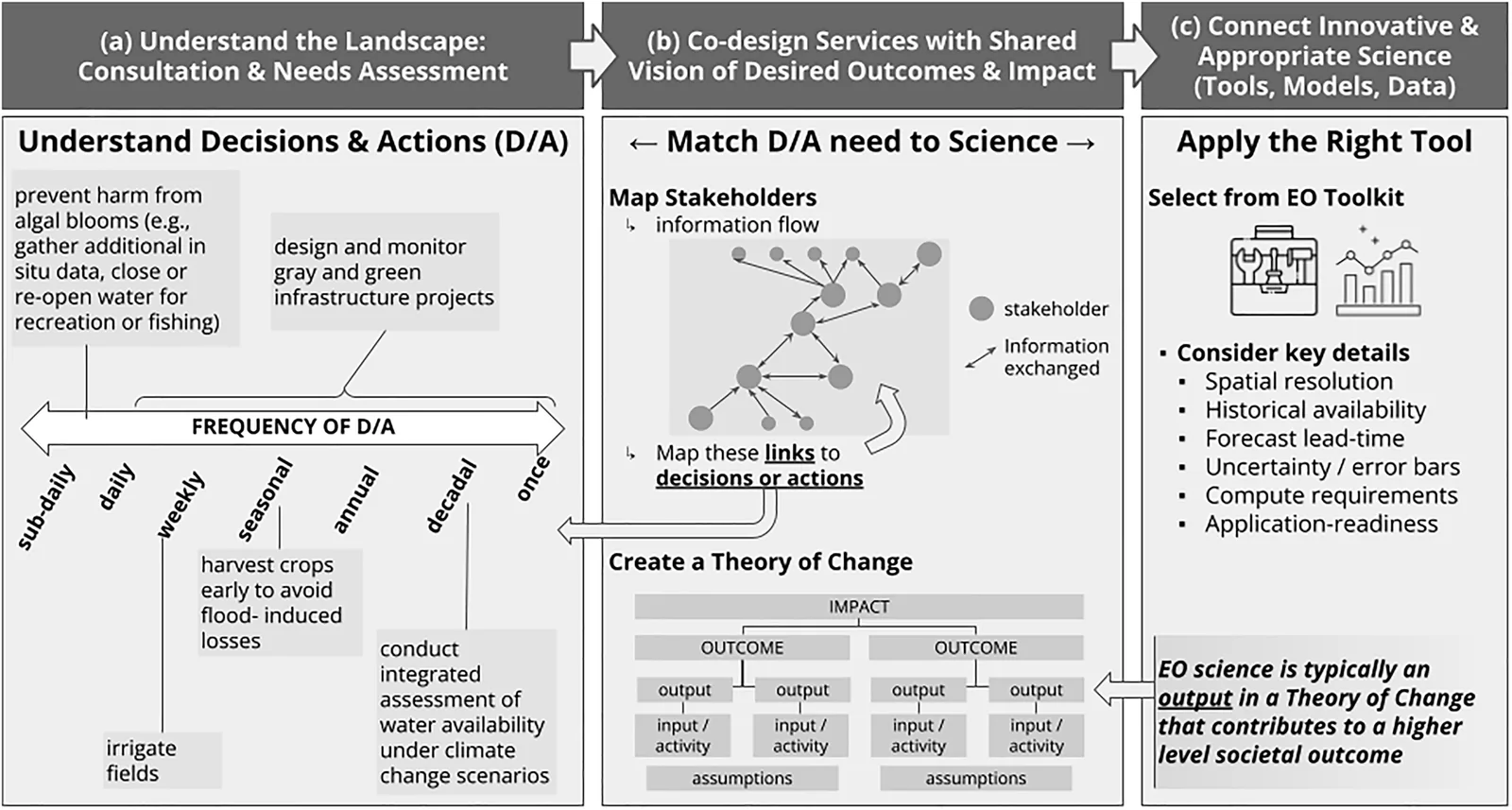
SERVIR’s Service Planning Approach for co-producing Earth observation services. The framework integrates (**a**) consultation and needs assessment, (**b**) co-design with shared vision of desired outcomes, and (**c**) alignment of appropriate EO science tools. This iterative process maps stakeholder decisions, defines theories of change, and applies fit-for-purpose scientific models to achieve actionable EO information. Adapted from^18^.

At the core of SERVIR’s methodology was the Service Planning Approach (SPA), a stepwise framework for transforming EO data into decision-support services. The SPA began with stakeholder consultations to identify priority decisions and associated information gaps; it then mapped those needs to potential EO products and analytical tools; finally, it guided iterative co-development and evaluation. This process required sustained dialogue rather than one-off training events or product launches. By embedding co-design at each stage, SERVIR avoided the common pitfall of technology transfer programs—delivering tools without institutional ownership^21,22^. The SPA thus served as a practical expression of adaptive governance, coupling flexibility in tool design with accountability to user needs (Fig. 2).

### Capacity and translation: building human infrastructure

Co-development depends on human intermediaries—individuals fluent in both science and local governance contexts. SERVIR invested heavily in training such intermediaries, known as “service leads” or “translators”, who could navigate between technical and policy communities. These individuals facilitated feedback loops, ensured that scientific outputs addressed specific decisions, and mentored national counterparts. Over time, this human infrastructure^23^ became a core asset of SERVIR’s sustainability: as personnel moved into government roles or other organizations, they carried the co-development ethos with them, seeding new collaborations and expanding the network’s reach.

### Evaluation and learning: institutional reflexivity

SERVIR’s partnership with USAID introduced a culture of monitoring, evaluation, and learning (MEL) rarely seen in science programs. Each service underwent systematic assessment of its usability, uptake, and influence on decisions. Beyond tracking outputs, these evaluations documented pathways of change—how EO information entered policy cycles, budget allocations, or community actions. The MEL framework created institutional reflexivity, allowing scientists, translators, and the entire network to learn from failures, adjust service design, and communicate impact in development terms. This systematic assessment defined the criteria for sustained institutional integration validated in this paper, focusing on policy uptake measured by the formal inclusion of EO information in national or regional decision systems (e.g., daily bulletins, SOPs, and budgetary influence demonstrated when EO information guides real resource allocation [e.g., water trucking decisions, budget planning, or infrastructure investment]).

Institutional ownership was evidenced by partners adopting co-produced services for long-term use, securing national funding to sustain services after external donor support ceased, and maintaining operations and collaborations beyond the conclusion of US Government support in 2025—demonstrating the durability of the institutional capacities fostered through co-development.

This iterative learning loop distinguished SERVIR from many other EO initiatives that focused solely on singular data accuracy or model validation exercises.

### Theoretical positioning

From a sustainability transitions perspective, SERVIR can be understood as a transition intermediary—a network that orchestrated multi-actor collaborations across knowledge, technology, and governance systems^18,19^. Its long-term impact lay not only in producing data services but in reshaping relationships among institutions. SERVIR demonstrated that co-development is most effective when embedded within boundary structures that institutionalize participation and learning. This conceptual framing positions SERVIR as both a practical and theoretical contribution to the emerging science of sustainability transitions and transformative learning^24,25^.

## Earth observations can become part of routine decision-making

**Fig. 3:**
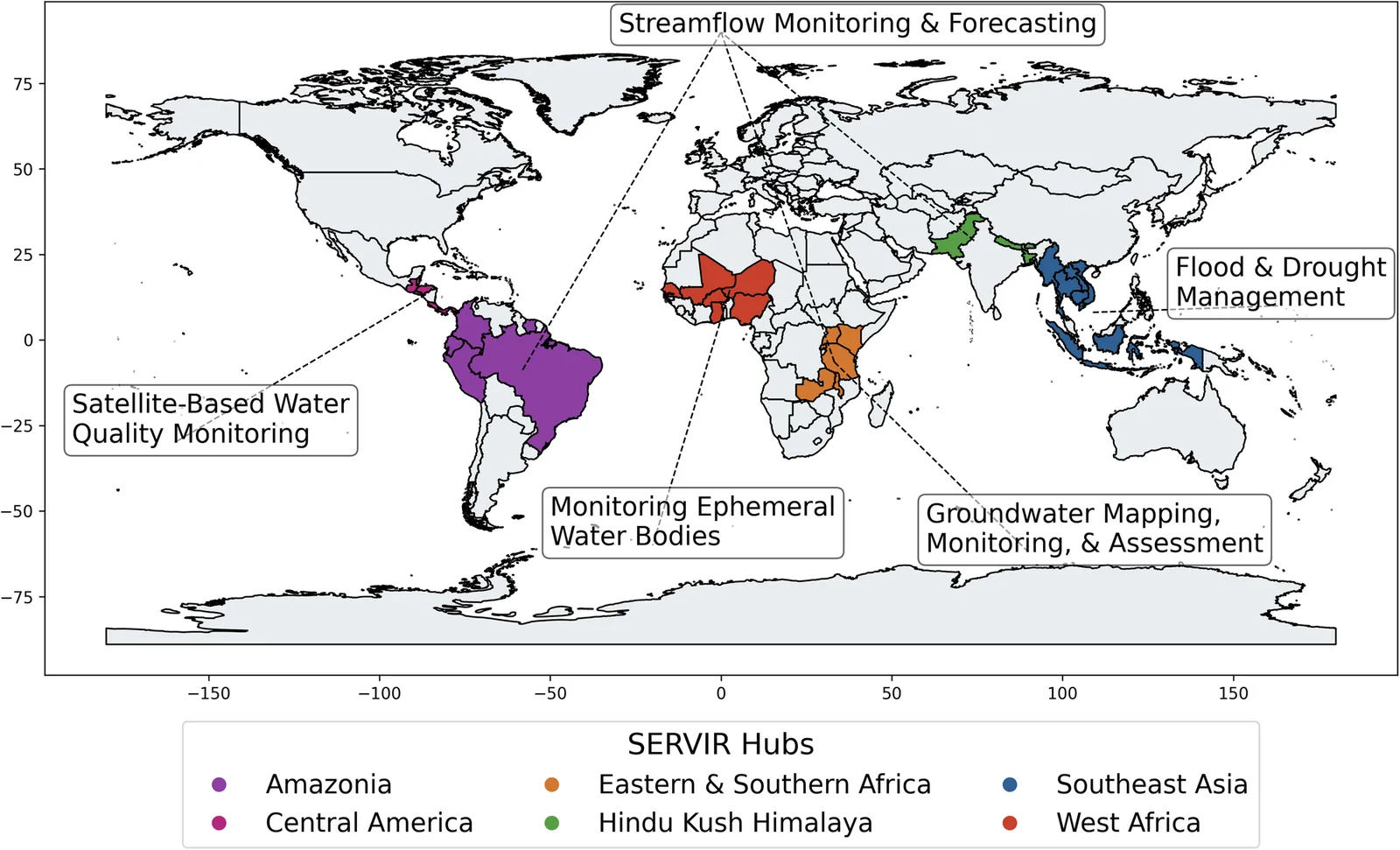
Global reach of SERVIR’s water portfolio across six regional hubs (2005-2025). SERVIR’s network of hubs across the Amazon, Central America, West Africa, Eastern & Southern Africa, the Hindu Kush Himalaya, and Southeast Asia co-developed services addressing streamflow forecasting, ephemeral water monitoring, groundwater management, and water quality assessment. Adapted from^18^.

Over two decades, SERVIR developed and institutionalized more than fifty EO-based services across six regional hubs (see Fig. 3). This synthesis demonstrates measurable sustainability outcomes by clustering examples into three functional domains where the co-development model produced sustained institutional integration: anticipatory action, water resilience, and environmental quality and health. Critically, SERVIR’s EO information was integrated into more than thirty-five national or regional decision systems, reaching millions of people through improved water and EO services. This reach was driven by building massive institutional capacity, providing training in remote sensing and geospatial skills to over 20,000 professionals across 2000 organizations in over 70 countries^26^. To examine early evidence of institutional continuity beyond initial deployment, Supplementary Table 1 summarizes representative satellite-enabled services, their decision contexts, evidence of iterative co-development, and, where documented, continued or transitioned use following the conclusion of donor support in 2025. Because the post-closure observation period remains short, these examples should not be interpreted as an exhaustive assessment of long-term sustainability across the full portfolio.

**SERVIR Legacy: By the Numbers**

- **6 Regional Hubs** across Africa, Asia, and Latin America
- **70+ Partner Countries** engaged in co-developing EO services
- **50+ Operational EO-based Services** for water, agriculture, and ecosystems
- **Over 2000 Institutions** collaborating through SERVIR’s network
- **Over 20,000 Professionals Trained** in geospatial and decision-support tools
- **EO Information Integrated** into 35+ national or regional institutional decision systems

Source: SERVIR program close-out report^26^. Figures represent cumulative global estimates across the 20-year program lifespan.

### From forecasts to anticipatory action

Anticipatory action refers to the capacity to foresee risks and act before hazards escalate into crises^27,28^. SERVIR’s regional hubs collaborated with hydrological and meteorological agencies to integrate satellite-enabled forecasts into operational early-warning systems. In the Hindu Kush Himalaya and the Mekong basins, for instance, flood forecasting services were co-designed with national and regional disaster authorities to supplement sparse gauge networks with remotely sensed rainfall and runoff data^29,30^. Through iterative testing and feedback, tools such as the Streamflow Prediction Tool (SPT) and the Flash Flood Prediction Tool (FFPT) became embedded in daily flood bulletins, providing lead times of up to ten days for major river systems. In East and Southern Africa, SERVIR’s collaboration with drought management authorities transformed seasonal forecasts into actionable planning tools. By coupling NASA’s land surface models with local monitoring data, SERVIR and partners contributed EO based information to the Drought Resilience Impact Platform (DRIP) that now feeds directly into national drought bulletins^31^. These bulletins guide water-trucking and contingency planning by agencies and humanitarian organizations, linking EO-based forecasts to real budgetary and logistical actions. Independent evaluations by USAID reported that SERVIR’s anticipatory services reached more than twenty national and regional institutions and informed the management of water resources affecting millions of people. These cases show that predictive science gains policy traction only when forecasts are co-owned by the institutions responsible for risk management—a central tenet of co-development.

### Water access and resilience: managing resources under uncertainty

A second adaptation function concerned water access—particularly in arid and semi-arid regions where groundwater and small water bodies are lifelines during drought. SERVIR’s hubs in Eastern and Western Africa co-developed decision-support systems to monitor groundwater^32,33^ and ephemeral water bodies^34,35^ using combined satellite and in situ data. In Kenya, SERVIR supported the National Drought Management Authority in mapping seasonal water availability for pastoral communities, integrating this information into monthly drought bulletins and coordination meetings. Similar systems in the Sahel combined optical imagery sources to monitor ephemeral ponds critical for livestock migration, promoting more inclusive water access among pastoral groups. These services exemplify co-development’s distributive dimension: they improved data quality while expanding access to decision-relevant information. By training local agencies and community-based organizations to interpret EO data, SERVIR lowered the barriers to EO information that otherwise would have been accessible to only highly technical users. The long-term outcome was a transition from reactive crisis management to proactive, inclusive water governance.

### Environmental quality: linking water quality and community action

SERVIR also applied EO science to environmental quality and public health—domains often marginalized in water management. In Central America, SERVIR’s collaboration with environmental authorities and universities around Lake Atitlán addressed recurrent harmful algal blooms threatening the local water supply and the economically important tourism sector. Satellite-based chlorophyll-a monitoring and spectral analysis, co-developed with local scientists, enabled early detection of bloom onset and guided mitigation actions. Following demonstration and validation, the monitoring service was institutionalized by the lake authority and integrated into public communication systems^36–38^. Comparable efforts in South and Southeast Asia linked EO-based sediment and land-degradation monitoring to reservoir management, allowing agencies to anticipate siltation risks and plan dredging. Across these initiatives, SERVIR’s unique value was institutional—not technological: it translated ecosystem monitoring into governance mechanisms that only informed regulation, enforcement, and local stewardship.

### Cross-cutting insights

Synthesizing across these domains reveals common mechanisms underpinning SERVIR’s sustainability. First, co-development created legitimacy through structural embedding. This mechanism influences institutional behavior by mapping EO tools directly onto existing operational workflows and protocols—such as established routines for issuing flash flood risk bulletins or drought bulletins. By integrating services as internal components of these existing workflows rather than introducing them as parallel, external tasks, the EO data ceases to be an isolated scientific product and becomes one of the many sources of information leading to operational decisions. This ensures that services align not just in theory, but in daily practice, with institutional priorities and policy cycles.

Second, embedding services within existing organizations with national and regional mandates—not temporary teams of consultants—ensured continuity beyond funding cycles. Third, iterative evaluation and learning cultivated reflexivity, enabling adaptation as conditions evolved. Finally, the regional hub network amplified innovations through transboundary learning and peer exchange.

Together, these patterns illustrate the effectiveness of a distributed learning ecosystem—an environment where science, governance, and society co-produce actionable intelligence for resource management and resilience.

## Long-Term Impact Depends on Institutional Capacity

Drawing from these insights allows for a synthesis of more general tenets of co-development and translational research.

### Trust, legitimacy, and boundary work

The literature on boundary organizations emphasizes three criteria for actionable knowledge: scientific credibility, political legitimacy, and salience to user needs^11,39^. Hub models can operationalize this triad. Locating hubs within respected regional institutions conferred legitimacy; collaboration with leading scientists ensured credibility; and participatory service design guaranteed salience. The interplay among these dimensions generated trust—a critical, if intangible, asset for sustainability transitions. Trust allows programs and networks to convene agencies that historically guarded their data, facilitating the creation of shared information systems for water and environmental management. Trust also extends across scales. Within each hub, local partners can often become champions of scientific methods, securing regional or national funding to sustain services after donor support ends and after science projects conclude. At the global level, the consistent partnership between science and donor agencies can exemplify boundary work within the donor and science communities themselves—aligning the priorities of an innovation-driven space agency with those of a development organization focused on resilience. This bi-agency collaboration is a strong model for sustained EO information services for myriad developmental and environmental outcomes.

### Institutional learning and reflexivity

Sustainability transitions depend on the capacity of institutions to learn from experimentation. Translators and boundary networks can institutionalize such learning through monitoring, evaluation, and learning (MEL) frameworks. Unlike conventional performance metrics that track only products, MEL systems can trace the pathways by which information influences decisions—what development evaluators term “use-based evidence”. By systematically documenting uptake, barriers, and unintended consequences, translators and boundary networks can turn each service into an experiment in adaptive governance. This reflexivity can foster an internal culture of learning. Regular exchanges brought together hub teams, and applied scientists to share lessons and recalibrate priorities. Failures need not be stigmatized but treated as learning opportunities—a practice rarely achieved in large public programs. This adaptive ethos can contribute to the longevity of the programs and generate lingering positive impacts.

### The role of human capital and network effects

The most enduring outcomes of successful sustainability infrastructures are not their data portals or algorithms but their people. Thousands of professionals trained through such networks tend to occupy positions in government ministries, regional organizations, and research institutions. These individuals constitute an epistemic community that continues to apply co-development principles beyond the program’s formal boundaries. Science agencies and development agencies play a pivotal role in maintaining an inclusive ecosystem throughout the network, and it is possible that their investments live on longer than their funding streams. By establishing governance structures, providing resources, and recognizing contributions, framing and funding agencies can ensure that both hub staff and scientists are rewarded for developing innovative, context-appropriate solutions. This support encourages the scaling and tailoring of solutions across diverse regional contexts, reinforcing collaboration, peer learning, and continuous improvement throughout networks. Networked structures illustrate how knowledge co-development scales through people as much as through platforms. They also create positive feedbacks: innovations developed in one hub often diffuse to others through peer learning, while common challenges stimulate collective problem-solving. This form of horizontal scaling—replication through tailoring to local contexts rather than replication through uniformity—proves critical for maintaining contextual relevance. It mirrors the dynamics of sustainability transitions more broadly, where change emerges from distributed innovation rather than top-down control.

### Challenges and adaptive learning in translational infrastructure

A robust sustainability strategy must account for the institutional and operational realities that accompany long-term development efforts. Over two decades, efforts to operationalize translational Earth observations encountered recurring constraints that highlight both the limits of co-development and important lessons for future initiatives.

A persistent challenge in translating Earth observations into sustained decision-making is the mismatch between project-based funding horizons and the long-term investments required to maintain operational environmental services. Many decision-support systems depend not only on satellite observations but also on complementary in situ monitoring networks, technical infrastructure, and skilled personnel that require continuous maintenance. For example, efforts to monitor groundwater demand in East Africa were periodically constrained by the degradation of the supporting observation network, including sensor vandalism, gateway malfunctions, and limited local maintenance capacity. Without sustained institutional and financial support, these components can deteriorate after project completion, limiting the durability of otherwise successful services. More broadly, these experiences illustrate that institutionalizing Earth observations depends as much on sustained organizational, financial, and governance capacity as on scientific innovation itself. Consequently, co-development should incorporate long-term institutional ownership, financing mechanisms, governance arrangements, and maintenance strategies from the outset rather than treating them as post-project considerations.

A second challenge concerns maintaining institutional continuity over the lifespan of operational services. As organizations evolve, priorities, staffing, and governance structures inevitably change, creating risks for preserving technical expertise, institutional memory, and long-term ownership. Operational use frequently declines when personnel trained to use a service transition to new roles or when government officials who championed its adoption are replaced. Experience across multiple SERVIR partnerships demonstrated that addressing these challenges requires cultivating regional champions, extending capacity development beyond individual practitioners, and maintaining comprehensive documentation so that knowledge is embedded within organizations rather than individuals. More broadly, these experiences suggest that institutional resilience depends on strengthening organizational learning, peer networks, and knowledge management systems that remain robust despite changing personnel and governance contexts. Translational capacity should therefore be viewed as an institutional capability that must be continually reinforced rather than an outcome achieved through a one-off training event.

Technological evolution presents both opportunities and implementation challenges for operational EO services. In several cases, scientific advances initially outpaced operational realities. For example, an early machine-learning model developed to forecast harmful algal blooms in Lake Atitlán proved unsuitable for routine decision support because the latency of the underlying satellite observations prevented timely daily alerts. Similarly, early ephemeral water monitoring systems in West Africa relied on short-range weather forecasts that provided insufficient lead time for pastoralists to safely reroute livestock. Subsequent transitions to cloud-based processing environments improved computational efficiency, expanded analytical capabilities, and reduced some of these latency constraints, but also introduced new governance, procurement, and data management challenges. Rather than treating operational services as static technological products, these experiences demonstrate the importance of adaptive governance capable of responding to evolving technologies, institutional priorities, and user needs. Sustained societal impact therefore depends on maintaining translational infrastructures that can evolve alongside changing scientific, technical, and governance environments rather than preserving any single technological solution.

Importantly, not all co-developed services transitioned successfully into sustained operational use. Some promising prototypes remained pilot demonstrations because long-term institutional ownership, financial commitments, or technical capacity could not be secured following initial project support. In other cases, services required substantial redesign as governance priorities, user requirements, or technological capabilities evolved. These experiences underscore that successful translation should not be evaluated solely by the deployment of new tools, but by the extent to which services become embedded within institutional routines, decision-making processes, and long-term governance arrangements. Recognizing these limitations provides a more realistic understanding of the time, resources, and institutional commitment required to translate Earth observations into durable societal outcomes.

### Lessons for sustainability transitions theory

Beyond their practical implications, the cases presented in Section 3 provide empirical insights into the institutional conditions under which EO innovations become embedded within operational governance systems. In doing so, they illustrate and enrich several propositions in sustainability transitions research by demonstrating how boundary organizations, co-development, translational expertise, and adaptive learning interact to support the long-term institutionalization of scientific knowledge. First, they demonstrate that boundary organizations can evolve into transition intermediaries- entities that coordinate multi-actor learning processes across scales. Second, they show that co-development can be institutionalized through formal frameworks like the Service Planning Approach, without losing its participatory essence. Third, they underscore the importance of donor-scientist partnerships as part of the transition system, not merely as external enablers.

By linking global EO infrastructures with local governance processes, translational partnerships and boundary organizations exemplify multi-level transition systems in which niche innovations (EO services) interact with institutional regimes (for example, national agencies and decision-making processes) and broader socio-environmental landscapes. Two decades of sustained programming and partnership provide a rare opportunity to observe institutional maturation—a feature often absent from short-term sustainability initiatives. Taken together, these cases suggest that the long-term societal impact of EO depends not only on technological innovation but also on the institutional arrangements that enable knowledge to be translated, sustained, and adapted over time. Rather than viewing boundary organizations, co-development, translational expertise, and adaptive learning as independent components, the cases examined here indicate that they function as an interconnected translational infrastructure through which EO innovations become embedded within operational governance systems. This perspective complements sustainability transitions research by highlighting the institutional processes that enable EO to generate durable societal impact.

## Future Earth Observation Systems Need Stronger Institutional Foundations

The conclusion of NASA and USAID support for SERVIR in 2025 marks not an end but a transformation of its functions into broader sustainability infrastructures. Its legacy—data systems, institutional networks, and governance models—now underpins a global transition toward open, inclusive, and digitally enabled adaptation. This section outlines how SERVIR’s experience can inform the design of next-generation adaptation systems, emphasizing four interrelated pathways: digital public goods, open science and AI, resilience finance alignment, and inclusive governance.

### Digital Public Goods for Planetary Resilience

One of SERVIR’s most profound contributions was the tailoring of interoperable, openly accessible data systems. Many of its tools—streamflow models, drought indices, and land-cover maps—are now embedded in global platforms such as GEOGloWS^40^. These resources exemplify the notion of digital public goods—open-source data and algorithms that can be adapted and repurposed by diverse actors to serve the common good. The next phase of sustainability science should treat such infrastructures as shared observational and monitoring assets, not technical by-products. This requires investment in standards, stewardship, and inclusive access, ensuring that the benefits of global data are distributed fairly across regions and communities.

### Open science, artificial intelligence, and co-development 2.0

SERVIR’s emphasis on co-development prefigured today’s calls for open and participatory science. The proliferation of cloud computing and artificial intelligence (AI) tools offers new opportunities to scale this ethos. Machine learning models trained on EO data can enhance forecasting accuracy and automate anomaly detection, while open APIs allow local developers to integrate these models into custom applications. However, as SERVIR’s experience showed, technology alone does not guarantee impact; it must be coupled with capacity-building and ethical governance. A future “Co-Development 2.0” model would combine AI-driven analytics with human-centered design, ensuring transparency, accountability, and inclusivity. SERVIR’s iterative engagement frameworks could serve as blueprints for embedding AI responsibly in EO information services.

### Aligning EO information with finance and policy

A persistent barrier to adaptation has been the disconnect between EO information and financial decision-making. SERVIR demonstrated that EO data services gain traction when tied to budgetary processes—such as drought contingency funds or agricultural insurance schemes. Future adaptation systems should institutionalize these linkages. For example, integrating EO-based risk metrics into disaster risk finance instruments could make resource allocation more transparent and performance-based. The growing momentum behind initiatives like the Global Adaptation Goal under the Paris Agreement presents an opportunity to mainstream SERVIR-like services within global finance architectures. Doing so would shift EO applications from pilot-scale demonstrations to core components of sustainable development investment.

### Inclusive and polycentric governance

SERVIR’s regional network model offers lessons for governing complex sustainability systems. Its polycentric structure—multiple centers of authority connected through shared standards, goals, and learning processes—proved resilient to political change and funding cycles. Future global adaptation infrastructures could adopt similar architectures, combining local autonomy with transnational coordination. In practice, this means empowering regional knowledge hubs, indigenous organizations, and local governments as co-owners of data ecosystems. The transition from hierarchical aid relationships to distributed knowledge partnerships is essential for local and regional resilience. SERVIR’s hubs illustrated that when local actors have genuine decision-making power over data and priorities, developmental and humanitarian outcomes are more durable and contextually appropriate.

### Measuring what matters: impact and accountability

The next generation of adaptation systems will need robust mechanisms for demonstrating societal impact. Monitoring, Evaluation, and Learning (MEL) frameworks such as SERVIR’s offer a valuable precedent, but future systems should go further—linking EO service outcomes to indicators of resilience, livelihoods, and ecosystem health. Advances in geospatial analytics and impact assessment methodologies can make such linkages more precise. Embedding accountability in open-data principles will help ensure that public investments in EO information infrastructure yield tangible public value. Taken together, these pathways define a vision for planetary resilience infrastructures—integrated networks of data, institutions, and communities co-producing knowledge for a rapidly changing world. SERVIR’s experience shows that such infrastructures are achievable when technology is in service of shared goals and when partnerships prioritize actionable outcomes over control.

## Institutions Matter as Much as Technology

The rapid expansion of EO capabilities has transformed our ability to monitor environmental change, yet translating these scientific advances into sustained societal impact remains an institutional challenge. Drawing on two decades of experience from SERVIR, this perspective uses the program as an illustrative example to examine how EO can become embedded within operational governance systems. Rather than presenting a program-specific retrospective, it synthesizes a set of transferable institutional characteristics—including trusted boundary organizations, structured co-development, translational expertise, and adaptive learning—that enable EO services to evolve from scientific innovations into sustained decision-support systems. The cases synthesized here demonstrate that long-term societal impact depends not only on advances in EO science, but also on the institutional arrangements that enable scientific knowledge to be translated, maintained, and adapted over time. Equally important, the challenges encountered—including funding discontinuities, institutional turnover, evolving technologies, and changing governance contexts—highlight that institutionalizing EO is an iterative process of organizational learning rather than a linear progression from research to operations. These experiences reinforce that successful EO initiatives require sustained investment in governance, partnerships, organizational capacity, and institutional ownership alongside continued scientific innovation. Although these insights are grounded in the SERVIR experience, they extend well beyond any single program. Governments, development organizations, and scientific institutions increasingly face the common challenge of translating rapidly expanding EO capabilities into sustained public value for climate adaptation, water security, disaster risk reduction, food systems, and ecosystem resilience. The institutional principles synthesized here provide a transferable framework for designing, evaluating, and sustaining future EO initiatives across diverse governance and development contexts. Ultimately, the enduring societal value of EO will depend less on generating ever-increasing volumes of environmental data than on strengthening the institutional conditions through which scientific knowledge becomes actionable, trusted, and enduring. By viewing EO as a long-term process of institutionalization rather than technology transfer alone, this perspective re-frames how sustainability science can understand, evaluate, and support the translation of EO into durable societal outcomes. In an era defined by accelerating global environmental change and uncertainty, the enduring value of EO will be determined not simply by the technologies we develop, but by our ability to build the partnerships, institutions, and communities that enable science to listen, learn, and act together with society.

## Supplementary information

Transparent Peer Review file

Supplementary Information
