# Supplementary material for: Institutional partnerships enable long-term use of Earth observations in water security and resilience
Source: Commun Sustain. 2026 Nov 9;1(1):168. doi: 10.1038/s44458-026-00178-9 (PMC13747811; doi:10.1038/s44458-026-00178-9)
Supplement: Supplementary file 1 [file 44458_2026_178_MOESM1_ESM.pdf]

nature portfolio

Peer Review File

Institutional partnerships enable long-term use of Earth observation in water security and resilience

Corresponding Author: Dr Chinmay Deval

This file contains all reviewer reports in order by version, followed by all author rebuttals in order by version.

Version 0:

Reviewer comments:

Reviewer #1

(Remarks to the Author)
I found this to be a timely and important Perspective on the role of Earth observation, co-development, and boundary institutions in translating science into operational water-security and resilience decisions. The manuscript's central argument is that SERVIR should be understood not simply as a technology-transfer programme, but as a form of "sustainability transition infrastructure" that links global EO capabilities with local and regional decision systems. This framing should be of interest to a wide audience including, but not limited to the water-security, disaster-risk, adaptation, and sustainability-transitions communities.

The paper's major claims are that SERVIR:

Bridged the usability gap between EO science and decision-making especially in low and middle-income countries.

Created durable institutional capacity through regional hubs, service planning, translators, and MEL systems;

Supported water resilience, and environmental-quality decisions; and offered design principles for future open and inclusive systems.

I thought the strongest contribution was in the value of institutional arrangements that make the data actionable.

In terms of novelty, I felt it was moderate. A lot of the ideas around co-development and knowledge co-production have already been published on extensively, and these are well cited in the paper. I think the paper would be stronger if it focused more on what SERVIR can teach us that the broader literature has not already shown.

In terms of being persuasive, I think the evidence needs to be strengthened. The paper makes strong claims about durability, transferability, policy uptake, budgetary influence, and the number of people reached, but it would be better to demonstrate this evidence than just suggesting it. Where are the methods and evidence, for example, on the estimated 10 million people reached?

It would be good to provide further information/evidence on how SERVIR is durable. After the USAID/donor support ended what is the evidence of continued use? Some specific examples would be helpful. What services have continued? How many did not? Why?

A summarsing table would help with service name/country/decision context/ data source/evidence of upatake/evidence of continued use/citation or source – or something along those lines.
I also noted that the MEL system talked about documenting failures, but there is little discussion in the paper about these. A 20 year program should have some useful studies on failures that would be interesting for a perspective of this nature.

Finally, the authors should add a note about their role in SERVIR, in short, the article should avoid reading like a programme close-out document.

The "SERVIR Legacy" box gives headline figures — six hubs, 60+ partner countries, 70+ operational EO services, 611

institutions, over 2,500 professionals trained, and 35+ institutional decision systems, but the source is only described as a SERVIR monitoring, evaluation and learning report. The report should be fully cited, publicly accessible if possible, and accompanied by definitions and methods.

It would also be helpful to distinguist between this paper and Deval et al. 2024 that appears to cover SERVIR's journey in EO for water-resources management and future pathways.

Line Specific comments:
41 "one of the pressing challenges" "one of the most pressing challenges"
56 "The accelerating global environmental change" "Accelerating global environmental change"
70 "From 2005 when it was established to 2025 when..." "From 2005, when it was established, to 2025, when..."
87–88 "learning, negotiation, actions and decision" "learning, negotiation, action and decision-making"
111 "Although, it is important to note" "Although it is important to note"
233 "enitre network" "entire network"
279 vs 48 Inconsistency: "60+ Partner Countries" vs "50 countries" earlier Check please
279 vs 292 Box says six hubs; Fig. 3 caption says five regional hubs but lists six Check please
286–287 MEL report named but not fully referenced Add ref
389 "resources management" "resource management"
414 "strong a model" "a strong model"
416–417 "track only the products" "track only products"
419 "information influence decisions" "information influences decisions"

427 "This reflexivity can fostered" "This reflexivity can foster" or "fostered"
449 "innovations developed in one hub often diffuses" "innovations developed in one hub often diffuse"
452 "prove critical" "proved critical" or "proves critical"
463 "they underscores" "they underscore"
469 "programming and partnering has" "programming and partnering have"
494 "shared observational, and monitoring assets" Remove ','
508 "human-centered" Please use either "human-centred" or "human-centered" consistently
529 "proved somewhat resilient" "proved resilient" or explain somewhat
556 "resislience" "resilience"

Reviewer #2

(Remarks to the Author)

Summary
This manuscript distills two decades of experience from the SERVIR program into a set of transferable lessons for translating Earth observation science into real-world impact in low- and middle-income countries. The authors articulate a compelling argument that the durability and reach of SERVIR's outcomes were driven less by technological innovation per se and more by sustained investment in human infrastructure: partnerships, regional institutions, capacity building, and co-development practices. The closing message, that "sustainable solutions emerge when science listens, learns, and acts together with society," captures the spirit of the contribution very well.

Overall Assessment
The manuscript is clearly written, well organized, and addresses a topic of high relevance to the international development, Earth observation, and translational science communities. Its contribution is twofold. First, it offers something close to a blueprint for future programs that aspire to bridge global scientific capacity with regional and local decision-making needs. Second, and equally important, it preserves part of the institutional history of SERVIR itself, a record that constitutes an important heritage for future generations of practitioners and program designers undertaking similar efforts. The emphasis on boundary organizations, reflexivity, and the gradual evolution from centralized data portals toward participatory service design is articulated thoughtfully and supported by the breadth of SERVIR's record. I particularly appreciated the framing of co-development as something that must be institutionalized rather than treated as an optional add-on; this is an important message for funders, host institutions, and implementing partners alike.

I recommend acceptance with minor revisions.

Main Comment
My one substantive suggestion concerns balance. The manuscript does an excellent job of identifying the practices and conditions that enabled SERVIR's successes, but it offers comparatively little reflection on the challenges, missteps, or approaches that did not work along the way. Two decades of operating at this scale almost certainly generated a rich body of experience about pitfalls to avoid, partnerships that did not succeed, services that failed to gain traction, transitions that proved more difficult than anticipated, or structural constraints (funding cycles, political turnover in host institutions, donor expectations, attribution and credit dynamics, sustainability gaps after handoff) that complicated the co-development model in practice.
I appreciate that the authors may have made a deliberate choice to keep the narrative forward-looking and constructive, and I recognize that a perspective piece is not the place for an exhaustive post-mortem. I raise this less as a required revision and more as a wish-list item, because I think the manuscript's value as a blueprint for future efforts would be meaningfully

enhanced by even a modest treatment of what to avoid alongside what to emulate. Programs that follow SERVIR may not inherit its institutional memory, and the candid lessons from two decades of practice are arguably as valuable as the affirmative ones. A short subsection, or even a paragraph integrated into the conclusion, identifying a handful of recurring challenges and how the program adapted (or failed to adapt) to them would strengthen the contribution considerably and give the blueprint metaphor more depth.

Minor Comments
1. There are several typos throughout e.g.:
a. “resislience” appears in the conclusion instead of “resilience.”
b. “enitre” in L237
I am not listing them all here. I am sure a careful review by the authors and journal’s technical editor will suffice to address this.
2. L268: “Over two decades, SERVIR developed and institutionalized more than seventy Earth observation (EO)-based services across six regional hubs”. . A reference to some of these services (relevant citations or links to services etc) would be nice to add here if available.
3. L320: “satellite-derived forecasts”. given that satellite observations can be near real time (at best), the term satellite-derived forecasts is not very accurate. Satellites cannot by themselves provide forecasts. Consider potentially rephrasing to satellite-enabled or something similar.

Efthymios Nikolopoulos, Ph.D.
Associate Professor
Rutgers University

Reviewer #3

(Remarks to the Author)
This is a timely and well-written manuscript addressing an important topic at the intersection of Earth observation, sustainability science, and governance. The authors provide a rich synthesis of experiences from the SERVIR program and make a convincing argument that long-term institutional partnerships, co-development, and translational infrastructures are essential for connecting Earth observation capacities with sustainability decision-making.
The manuscript is particularly valuable in moving beyond purely technical discussions of EO applications and emphasizing governance processes, institutional learning, and science-policy interaction. The practical experience reflected in the paper is substantial and highly relevant.
My main concern is that the paper currently reads more as a synthesis or legacy narrative centered on the SERVIR program itself than as a broader conceptual contribution to sustainability governance or Earth observation science. SERVIR often becomes the subject of the paper, rather than serving as an example supporting a more generalizable framework or argument.
Related to this, several of the central themes, such as co-development, trust-building, institutional partnerships, and translational science, are important but not entirely new to the sustainability transitions and boundary organization literature. The manuscript would therefore benefit from more clearly articulating what conceptual advance is being made beyond demonstrating the success and evolution of SERVIR.
I would encourage the authors to further elevate the paper from a program-centered narrative toward a more generalized framework for how translational infrastructures support governance transformation. In particular, clarifying the mechanisms through which these approaches influence institutional behavior and decision-making would strengthen the manuscript considerably.

Version 1:

Reviewer comments:

Reviewer #1

(Remarks to the Author)
Thank you for the opportunity to review the revised manuscript. The authors have done a strong job addressing the main comments, and the paper is now clearer, better evidenced, and more balanced. I am happy to recommend publication, subject to any final editorial and production checks.

Reviewer #2

(Remarks to the Author)
The revised manuscript has improved substantially. I particularly appreciated section 4.4 Challenges and Adaptive Learning in Translational Infrastructure. The authors provided a detailed and adequate, In my opinion, response to reviewer's comments. I have no additional comments and I recommend publishing the revised version of the manuscript.

Reviewer #3

(Remarks to the Author)

I have carefully reviewed the revised manuscript and the authors' responses to my previous comments. I appreciate the substantial efforts made by the authors in revising the manuscript.

In my previous review, I suggested that the manuscript should move beyond a SERVIR-centered narrative and more clearly articulate the broader and transferable lessons that can be derived from its two decades of experience. I am pleased to see that the revised manuscript has addressed this concern effectively. In particular, the strengthened conceptual framing of translational infrastructures and institutional capabilities, together with the additional discussion of challenges and limitations, has significantly improved the manuscript and enhanced its broader relevance.

Overall, the authors have adequately addressed my previous concerns, and the revised manuscript is clearer, more balanced, and more compelling as a Perspective. I have no further substantive comments and recommend acceptance.

Open Access This Peer Review File is licensed under a Creative Commons Attribution 4.0 International License, which permits use, sharing, adaptation, distribution and reproduction in any medium or format, as long as you give appropriate credit to the original author(s) and the source, provide a link to the Creative Commons license, and indicate if changes were made.
In cases where reviewers are anonymous, credit should be given to 'Anonymous Referee' and the source.
The images or other third party material in this Peer Review File are included in the article's Creative Commons license, unless indicated otherwise in a credit line to the material. If material is not included in the article's Creative Commons license and your intended use is not permitted by statutory regulation or exceeds the permitted use, you will need to obtain permission directly from the copyright holder.
To view a copy of this license, visit https://creativecommons.org/licenses/by/4.0/

Reviewers' comments:

Reviewer #1 (Remarks to the Author):
I found this to be a timely and important perspective on the role of Earth observation, codevelopment, and boundary institutions in translating science into operational water-security and resilience decisions. The manuscript's central argument is that SERVIR should be understood not simply as a technology-transfer programme, but as a form of "sustainability transition infrastructure" that links global EO capabilities with local and regional decision systems. This framing should be of interest to a wide audience including, but not limited to the water-security, disaster-risk, adaptation, and sustainability-transitions communities.

The paper's major claims are that SERVIR:

- Bridged the usability gap between EO science and decision-making especially in low and middle-income countries.
- Created durable institutional capacity through regional hubs, service planning, translators, and MEL systems;
- Supported water resilience, and environmental-quality decisions; and offered design principles for future open and inclusive systems.

I thought the strongest contribution was in the value of institutional arrangements that make the data actionable. In terms of novelty, I felt it was moderate. A lot of the ideas around co-development and knowledge co-production have already been published on extensively, and these are well cited in the paper. I think the paper would be stronger if it focused more on what SERVIR can teach us that the broader literature has not already shown.

We deeply appreciate this insightful critique. We agree that merely advocating for co-development is no longer novel. In response, we have pivoted the Introduction and Discussion sections to explicitly analyze how specific institutional architectures—what we term "translational infrastructures"—are structured to make Earth observation data actionable. Rather than reiterating general co-production theory, we focus on the specific organizational mechanisms (such as structural embedding within existing workflows and the management of hardware decay) that the broader literature frequently overlooks.

Key Changes: Reframed Section 1 (Introduction) and Section 5 (Discussion) to emphasize structural mechanisms and generalizable design principles rather than general program history.

In terms of being persuasive, I think the evidence needs to be strengthened. The paper makes strong claims about durability, transferability, policy uptake, budgetary influence, and the number of people reached, but it would be better to demonstrate this evidence than just suggesting it. Where are the methods and evidence, for example, on the estimated 10 million people reached?

The reviewer was entirely correct to question the spatial methodology of the "10 million people" population estimate. Because generating a methodologically rigorous spatial estimate for global population reach carries high uncertainty, we have removed this unverified claim. Instead, we have grounded our evidence in the exact, verifiable programmatic metrics detailed in the official 20-year global close-out report (Irwin et al., 2025). The manuscript and the "Legacy Box" now highlight verifiable capacity-building metrics, including over 20,000 professionals trained across 2,000 organizations in over 70 countries.

Key Changes: Removed the "10 million" claim in Section 4; updated the "SERVIR Legacy" box and footnote to reflect cumulative 20-year close-out metrics backed by formal citation.

It would be good to provide further information/evidence on how SERVIR is durable. After the USAID/donor support ended, what is the evidence of continued use? Some specific examples would be helpful. What services have continued? How many did not? Why? A summarizing table would help with service name/country/decision context/ data source/evidence of upatake/evidence of continued use/citation or source – or something along those lines.

Thank you for this helpful suggestion. To address this comment comprehensively, we have compiled a dedicated **Supplementary Table 1** that provides a representative sample (rather than an exhaustive inventory) of satellite-enabled services that have demonstrated continued institutional use following the end of USAID/donor support. The table summarizes each service, its country or geography of implementation, the decision context it supports, the thematic area, the underlying data source, evidence of institutional uptake, evidence of continued operational use after donor funding ended, and the corresponding citation or URL documenting the evidence.

We have also added a direct callout to Supplementary Table 1 in Section 3 of the manuscript to guide readers to these examples. While the table does not quantify all services that have or have not continued, it provides concrete evidence of the durability of representative SERVIR-supported services across multiple countries and decision contexts, illustrating sustained institutional adoption beyond the donor-funded implementation period.

Key Changes: Added an explicit text reference in Section 4 directing readers to Supplementary Table 1, which details post-handoff durability across representative regional services

I also noted that the MEL system talked about documenting failures, but there is little discussion in the paper about these. A 20-year program should have some useful studies on failures that would be interesting for a perspective of this nature.

We strongly agree that documenting operational friction is essential for rigorous sustainability transitions research. We have significantly expanded our dedicated subsection on operational hurdles to detail specific, concrete missteps and limitations encountered over the 20-year record. This includes analyzing the physical decay of ground sensor networks due to vandalism

and maintenance gaps in East Africa, data latency mismatches that paralyzed real-time machine-learning alerts in Lake Atitlán, insufficient forecast lead-times for pastoralist decision-making in West Africa, and the vulnerability of institutional memory to high staff turnover.

Key Changes: Expanded Section 4.4 (Challenges and Adaptive Learning in Translational Infrastructure) to incorporate concrete examples of technical, physical, and institutional failures.

Finally, the authors should add a note about their role in SERVIR, in short, the article should avoid reading like a programme close-out document.

To ensure the manuscript maintains an objective, academic tone rather than reading as a program report, we have deliberately shifted the framing away from self-referential program reporting toward an analytical evaluation of an empirical record.

Key Changes: Ensured the text consistently analyzes the dataset from a scholarly perspective.

The "SERVIR Legacy" box gives headline figures — six hubs, 60+ partner countries, 70+ operational EO services, 611 institutions, over 2,500 professionals trained, and 35+ institutional decision systems, but the source is only described as a SERVIR monitoring, evaluation and learning report. The report should be fully cited, publicly accessible if possible, and accompanied by definitions and methods.

We have updated the source citation for the legacy box to officially reference the cumulative 20-year global program close-out report (irwin2025servir), replacing the point-in-time CY2023 figures with verified cumulative close-out metrics.

Key Changes: Updated the Legacy Box footnote in Section 4 to reflect the 20-year program close-out report and cited it appropriately in the bibliography.

It would also be helpful to distinguish between this paper and Deval et al. 2024 that appears to cover SERVIR's journey in EO for water-resources management and future pathways.

We appreciate the reviewer raising this distinction. We have added an explicit statement in the Introduction clarifying that while works such as Deval et al. (2024) comprehensively detail specific technical Earth observation tools and hydrologic applications, this Perspective isolates the institutional arrangements, boundary-spanning mechanisms, and governance structures required to make those tools actionable.

Key Changes: Added an explicit methodological clarification in Section 1 (Introduction).

Line Specific comments:
41 "one of the pressing challenges" "one of the most pressing challenges"

56 “The accelerating global environmental change” “Accelerating global environmental change”
70 “From 2005 when it was established to 2025 when...” “From 2005, when it was established, to 2025, when...”
87–88 “learning, negotiation, actions and decision” “learning, negotiation, action and decisionmaking”
111 “Although, it is important to note” “Although it is important to note”
233 “enitre network” “entire network”
279 vs 48 Inconsistency: “60+ Partner Countries” vs “50 countries” earlier Check please
279 vs 292 Box says six hubs; Fig. 3 caption says five regional hubs but lists six Check please
286–287 MEL report named but not fully referenced Add ref
389 “resources management” “resource management”
414 “strong a model” “a strong model”
416–417 “track only the products” “track only products”
419 “information influence decisions” “information influences decisions”
427 “This reflexivity can fostered” “This reflexivity can foster” or “fostered”
449 “innovations developed in one hub often diffuses” “innovations developed in one hub often diffuse”
452 “prove critical” “proved critical” or “proves critical”
463 “they underscores” “they underscore”
469 “programming and partnering has” “programming and partnering have”
494 “shared observational, and monitoring assets” Remove ‘,’
508 “human-centered” Please use either “human-centred” or “human-centered” consistently
529 “proved somewhat resilient” “proved resilient” or explain somewhat
556 “resislience” “resilience”

Thank you. We have meticulously addressed all line-specific corrections.

Reviewer #2 (Remarks to the Author):

Summary
This manuscript distills two decades of experience from the SERVIR program into a set of transferable lessons for translating Earth observation science into real-world impact in low- and middle-income countries. The authors articulate a compelling argument that the durability and reach of SERVIR’s outcomes were driven less by technological innovation per se and more by sustained investment in human infrastructure: partnerships, regional institutions, capacity building, and co-development practices. The closing message, that “sustainable solutions emerge when science listens, learns, and acts together with society,” captures the spirit of the contribution very well.

Overall Assessment
The manuscript is clearly written, well organized, and addresses a topic of high relevance to the international development, Earth observation, and translational science communities. Its contribution is twofold. First, it offers something close to a blueprint for future programs that aspire to bridge global scientific capacity with regional and local decision-making needs.

Second, and equally important, it preserves part of the institutional history of SERVIR itself, a record that constitutes an important heritage for future generations of practitioners and program designers undertaking similar efforts. The emphasis on boundary organizations, reflexivity, and the gradual evolution from centralized data portals toward participatory service design is articulated thoughtfully and supported by the breadth of SERVIR's record. I particularly appreciated the framing of co-development as something that must be institutionalized rather than treated as an optional add-on; this is an important message for funders, host institutions, and implementing partners alike.

I recommend acceptance with minor revisions.

Main Comment

My one substantive suggestion concerns balance. The manuscript does an excellent job of identifying the practices and conditions that enabled SERVIR's successes, but it offers comparatively little reflection on the challenges, missteps, or approaches that did not work along the way. Two decades of operating at this scale almost certainly generated a rich body of experience about pitfalls to avoid, partnerships that did not succeed, services that failed to gain traction, transitions that proved more difficult than anticipated, or structural constraints (funding cycles, political turnover in host institutions, donor expectations, attribution and credit dynamics, sustainability gaps after handoff) that complicated the co-development model in practice.

I appreciate that the authors may have made a deliberate choice to keep the narrative forward looking and constructive, and I recognize that a perspective piece is not the place for an exhaustive post-mortem. I raise this less as a required revision and more as a wish-list item, because I think the manuscript's value as a blueprint for future efforts would be meaningfully enhanced by even a modest treatment of what to avoid alongside what to emulate. Programs that follow SERVIR may not inherit its institutional memory, and the candid lessons from two decades of practice are arguably as valuable as the affirmative ones. A short subsection, or even a paragraph integrated into the conclusion, identifying a handful of recurring challenges and how the program adapted (or failed to adapt) to them would strengthen the contribution considerably and give the blueprint metaphor more depth.

We sincerely thank Reviewer 2 for this constructive feedback and positive assessment. We fully agree that highlighting friction and missteps makes the blueprint for future initiatives much more robust. As noted in our response to Reviewer 1, we substantially expanded Section 5.4 (Challenges and Adaptive Learning in Translational Infrastructure) to openly discuss real-world hurdles—such as sensor vandalism and maintenance gaps in East Africa, data latency challenges in Lake Atitlán, forecast timing mismatches in West Africa, and institutional loss of memory due to staff turnover.

Key Changes: Expanded Section 4.4 to balance operational successes with candid reflections on technical, physical, and institutional challenges.

Minor Comments

1. There are several typos throughout e.g.:
a. “resislience” appears in the conclusion instead of “resilience.”
b. “enitre” in L237
I am not listing them all here. I am sure a careful review by the authors and journal’s technical editor will suffice to address this.
2. L268: “Over two decades, SERVIR developed and institutionalized more than seventy Earth observation (EO)-based services across six regional hubs”. . A reference to some of these services (relevant citations or links to services etc) would be nice to add here if available.
3. L320: “satellite-derived forecasts”. given that satellite observations can be near real time (at best), the term satellite-derived forecasts is not very accurate. Satellites cannot by themselves provide forecasts. Consider potentially rephrasing to satellite-enabled or something similar.

We have corrected all identified typos ("resilience", "entire") and systematically updated instances of "satellite-derived forecasts" to "satellite-enabled forecasts" to reflect that satellites provide data streams rather than standalone meteorological forecasts. Furthermore, we ensured key operational services are robustly referenced in the text and cataloged in Supplementary Table 1.

Key Changes: Fixed typos, updated terminology to "satellite-enabled forecasts" in Section 4.1, and ensured comprehensive service referencing.

Reviewer #3 (Remarks to the Author):
This is a timely and well-written manuscript addressing an important topic at the intersection of Earth observation, sustainability science, and governance. The authors provide a rich synthesis of experiences from the SERVIR program and make a convincing argument that long-term institutional partnerships, co-development, and translational infrastructures are essential for connecting Earth observation capacities with sustainability decision-making.

The manuscript is particularly valuable in moving beyond purely technical discussions of EO applications and emphasizing governance processes, institutional learning, and science-policy interaction. The practical experience reflected in the paper is substantial and highly relevant.
My main concern is that the paper currently reads more as a synthesis or legacy narrative centered on the SERVIR program itself than as a broader conceptual contribution to sustainability governance or Earth observation science. SERVIR often becomes the subject of the paper, rather than serving as an example supporting a more generalizable framework or argument.
Related to this, several of the central themes, such as co-development, trust-building, institutional partnerships, and translational science, are important but not entirely new to the sustainability transitions and boundary organization literature. The manuscript would therefore benefit from more clearly articulating what conceptual advance is being made beyond demonstrating the success and evolution of SERVIR.
I would encourage the authors to further elevate the paper from a program-centered narrative toward a more generalized framework for how translational infrastructures support governance transformation.

We deeply appreciate this core critique, which directly aligns with the Editor's guidance. We have reframed the manuscript to elevate it from a program legacy narrative to a broader conceptual contribution. We explicitly frame the work around four interacting institutional capabilities (boundary organizations, structured co-development, translational expertise, and adaptive learning) that constitute a transferable architecture for sustainability transitions.

Key Changes: Revised the Abstract, Introduction, and Conclusion to explicitly state the conceptual advance and frame SERVIR as an empirical case study for translational infrastructures.

In particular, clarifying the mechanisms through which these approaches influence institutional behavior and decision-making would strengthen the manuscript considerably.

This is a vital conceptual enhancement. We have expanded our cross-cutting insights to explicitly define how this model alters institutional behavior through the mechanism of "structural embedding." We clarify that behavior changes because Earth observation tools are mapped directly onto existing operational workflows and protocols (such as established routines for issuing flash flood risk bulletins or drought bulletins). By integrating services as internal components of these existing workflows rather than introducing them as parallel, external tasks, the data ceases to be an isolated scientific product and becomes one of the many sources of information leading to operational decisions.

Key Changes: Revised Section 3.4 (Cross-Cutting Insights) to explicitly unpack "structural embedding" and its operational mechanics.

We sincerely thank the reviewers for their careful evaluation of the revised manuscript and for their positive and constructive feedback. We are grateful that the revisions have satisfactorily addressed the concerns raised during the previous round of review.

Reviewer #1

We thank the reviewer for their positive assessment of the revised manuscript and for noting the improvements in clarity, evidentiary support, and balance. We appreciate the recommendation for publication and have carefully completed the remaining editorial amendments and final production checks requested by the Editor.

Reviewer #2

We thank the reviewer for their thoughtful assessment and for specifically recognizing the strengthened discussion in Section 4.4, Challenges and Adaptive Learning in Translational Infrastructure. We appreciate the reviewer's confirmation that the previous comments have been adequately addressed and their recommendation for publication. No further substantive changes were requested.

Reviewer #3

We sincerely thank the reviewer for their careful reassessment of the manuscript and for recognizing the strengthened conceptual framing around translational infrastructures and institutional capabilities, as well as the expanded discussion of challenges and limitations. We appreciate the reviewer's conclusion that the revised manuscript now more effectively communicates the broader and transferable lessons arising from the 20-year empirical record. We are grateful for the recommendation for acceptance.

Final note

In response to the Editor's final requests, we have incorporated the proposed title, revised the Abstract, updated the main section headings to make the central claims more declarative, and carefully reviewed the final manuscript files for consistency and production readiness.
