# Supplementary material for: Institutional partnerships enable long-term use of Earth observations in water security and resilience
Source: Commun Sustain. 2026 Nov 9;1(1):168. doi: 10.1038/s44458-026-00178-9 (PMC13747811; doi:10.1038/s44458-026-00178-9)
Supplement: Supplementary file 2 [file 44458_2026_178_MOESM2_ESM.pdf]

Sr. No.	Title	Start Date	Description	Countries
1	Landslide Hazard Assessment and Forecasting System for SERVIR Mesoamerica and HKH Regions	2012	This project uses a satellite-based landslide hazard assessment and forecasting products to identify areas susceptible to landslides.	Pakistan, Nepal, Bhutan, Bangladesh, India, El Salvador, Panama
2	Satellite Radar-derived Virtual Rain and Stream Gauge Data Service	2016	This service provides near real-time rainfall and stream height data from publicly available satellite measurements by creation of a virtual network of rain gauges and stream gauges at points widely distributed over the entire Lower Mekong Region.	Cambodia, Laos, Myanmar, Thailand, Vietnam
3	Regional Drought Information System	2016	This information system provides drought indices and crop forecast information (via a public web portal) to inform decision-making among officials in the Lower Mekong region given anticipated scenarios of the availability of water in streams and soils.	Cambodia, Laos, Myanmar, Thailand, Vietnam
4	Development and Management of Groundwater Analysis and Information System in Niger	2019	The Development and Management of Groundwater Analysis and Information System in Niger is a platform to improve and make available information on groundwater in Maradi and Zinder. This service consists of developing a geospatial model that combines sensor data with satellite imagery to provide information about groundwater resources which may include recharge and the volume of the groundwater. The service will enable the government and local communities to better monitor groundwater resources and make appropriate decisions related to irrigation and agriculture.	Niger

Sr. No.	Title	Start Date	Description	Countries
5	Enhancing Flood Early Warning Services in the Hindu Kush Himalaya Region	2018	SERVIR HKH, with the technical support of Brigham Young University, developed three products as part of the Enhancing Flood Early Warning service. 1) The service developed a streamflow prediction tool that incorporates all primary and secondary rivers in the HKH region, based on European Centre for Medium-Range Weather Forecasts (ECMWF) ensemble forecasting. 2) Using the precipitation forecast from the High-Impact Weather Assessment Toolkit (HIWAT), a streamflow forecasting system was developed to support flash flood early warning in small local rivers in Bangladesh and Nepal. This system provides a lead time of 54 hours.A mobile application, ,ÄúPrakop Alert,,Äù was recently launched on the Play Store. 3) With a request from Flood Forecasting & Warning Centre (FFWC) in Bangladesh, a customized flash flood prediction model using the HIWAT data was developed for the Meghna River Basin using the J2000 hydrological model.	Bangladesh, Bhutan, Nepal
6	Flood Inundation and Flood Occurrence Mapping	2020	Floods are a recurring event in the HKH region that often have disastrous consequences. SERVIR HKH and University of Alaska Fairbanks, along with regional and national level partners have developed the methodology and workflow for inundation extent and flood depth mapping. Through this collaboration they developed the Flood Inundation Mapping Tool (aka HydroSAR), a cloud-based system developed to map flood areas in Bhutan, Bangladesh, Nepal, and the northeast part of India.	Bangladesh, Bhutan, Nepal
7	Flood Monitoring in Specific Basins in Colombia	2021	This flood monitoring service utilizes Google Earth Engine and the available Landsat and Sentinel data collections, spanning from 2014 to the present date, to determine water surface areas within specific date ranges.	Colombia
8	Improving Resilience and Reducing Risk of Extreme Hydrological Events	2019	The service provides stakeholders in the Amazon Basin region with improved historical water information and a flood forecasting ability, including more accurate information about timing, magnitude, and impact, to increase their understanding of historical streamflow and to support greater resiliency to flood disasters.	Brazil, Colombia, Ecuador, Peru

Sr. No.	Title	Start Date	Description	Countries
9	Improving the Mekong River Commission's Regional Flood Forecasting	2017	Through this service, SERVIR Mekong supported the MRC to use new-generation satellite-derived precipitation products to increase flood forecast accuracy.	Cambodia, Laos, Thailand, Vietnam
10	Monitoring Ephemeral Water Bodies in Ferlo, Senegal	2017	This service is a web-based platform for assessing water availability in all known ponds, thus facilitating water management in arid areas such as the Ferlo Region of Senegal. The platform, known as WENDOU (Water ENvironment Dashboard for Observation in support of Users in Ferlo, Senegal) uses Earth observation data from Landsat 7 and 8 and Sentinel 2 to identify water availability.	Senegal
11	Supporting Better Riverine and Flash Flood Forecasting for the Lower Mekong	2019	This service improves riverine flood forecasts and enhances flash flood guidance monitoring in the region. The service provides decision-support tools to the Mekong River Commission (MRC) and governments in the region with a range of applications such as: flood forecasting and mapping, early warning systems and river basin management alongside capacity building activities.	Cambodia, Laos, Myanmar, Thailand, Vietnam
12	Satellite-based monitoring of lakes	2010	This project facilitated co-development of products related to monitoring priority water bodies in Guatemala, using both remote sensing and field data, with Guatemala's Ministry of Environment and Natural Resources (MARN) and Watershed Authority staff.	Guatemala
13	Developing Dynamic Web-GIS Based Early Warning System for the Communities at Landslide Risks in Chittagong Metropolitan Area	2014	The application will use geospatial information and spatial statistics study relationships among land cover change, rainfall, climate and landslide disaster and develop a dynamic model for land slide early warning in Bangladesh.	Bangladesh
14	GEOGLOWS implemented and validated as official input for national water resource management (Gutierrez 2025)	2024	The GEOGLOWS Streamflow Forecasting initiative leverages advanced hydrological models and high-resolution global meteorological data to provide accurate, real-time forecasts of river discharge for millions of stream segments worldwide. The system's improved data services, enhanced visualization tools, and robust analytical capabilities enable users to access, analyze, and apply streamflow data efficiently for a wide range of applications, from flood management and early warning systems to water resource planning. With continuous updates and community engagement, GEOGLOWS ensures that its forecasting services remain at the forefront of hydrological science and technology.	Bangladesh, Belize, Bhutan, Brazil, Colombia, Dominican Republic, Ecuador, Honduras, India, Malawi, Nepal, Panama,

Sr. No.	Title	Start Date	Description	Countries
15	Expansion of CREST Hydrologic Modeling Tool	2013	This tool provides water managers with information about recent streamflow amounts that can enable better decision-making regarding water resources, floods, and agriculture.	Bhutan, Kenya, Rwanda, Uganda, Namibia
16	CREST Streamflow Viewer - Eastern Africa and Bhutan	2013	This application provides a customized online viewer to visualize CREST Hydrologic Model information on precipitation and streamflow output to assess likelihood of flooding.	Bhutan, Kenya, Rwanda, Uganda, Namibia, Tanzania

Sr. No.	Thematic Area	Hub	URL	Notes
1	Water Resources, Weather Resilience, Disasters	SERVIR Mesoamerica/ SERVIR HKH	https://servir.adpc.net/tools/lhasa_detail.html	
2	Water Resources, Weather Resilience, Disasters	SERVIR Mekong	https://vrsg-servir.adpc.net/	
3	Water Resources, Disasters	SERVIR Mekong	https://seadw-servir.adpc.net/	
4	Water Resources	SERVIR WA, SERVIR SCO	https://apps.geoglows.org/grace-groundwater	

Sr. No.	Thematic Area	Hub	URL	Notes
5	Weather Resilience, Water Resources, Disasters	SERVIR HKH	http://tethys.icimod.org/apps/hiwatbd/<br>http://tethys.icimod.org/apps/hiwatnp/<br>http://tethys.icimod.org/apps/hiwatbt/	
6	Weather Resilience, Water Resources, Disasters	SERVIR HKH	https://geoapps.icimod.org/FloodInundation/	
7	Weather Resilience, Water Resources, Disasters	SERVIR Amazonia	https://inamhi.geoglows.org/apps/	
8	Water Resources, Weather Resilience	SERVIR Amazonia	https://inamhi.geoglows.org/; https://ideam.geoglows.org/apps/	

Sr. No.	Thematic Area	Hub	URL	Notes
9	Weather Resilience, Water Resources, Disasters	SERVIR Mekong	http://ffp.mrcmekong.org:8000/bulletin/	
10	Agriculture & Food Security, Water Resources	SERVIR WA, SERVIR SCO	https://wendou.cse.sn/	
11	Weather Resilience, Water Resources, Disasters	SERVIR SEA	https://servir.adpc.net/tools/rat_detail.html	
12	Water Resources, Disasters	SERVIR Mesoamerica	https://freshwater.net/home	
13	Weather Resilience, Water Resources, Disasters	SERVIR HKH	https://www.landslidebd.com/ctg-warning-system/	
14	Water Resources, Disasters	SERVIR HKH, SERVIR Amazonia, SERVIR Global	https://www.geoglows.org/	

Sr. No.	Thematic Area	Hub	URL	Notes
15	Agriculture & Food Security, Disasters, Water Resources, Weather Resilience	SERVIR E&SA/SERVIR HKH	https://www.ou.edu/coe/labs/hydros/crest	
16	Agriculture & Food Security, Disasters, Water Resources, Weather Resilience	SERVIR E&SA/SERVIR HKH	http://tethys.icimod.org/apps/hkhbasins/	Evolved into Streamflow Prediction Tool (SPT)
